# Paralytic Shellfish Toxins in Surf Clams *Mesodesma donacium* during a Large Bloom of *Alexandrium catenella* Dinoflagellates Associated to an Intense Shellfish Mass Mortality

**DOI:** 10.3390/toxins11040188

**Published:** 2019-03-29

**Authors:** Gonzalo Álvarez, Patricio A. Díaz, Marcos Godoy, Michael Araya, Iranzu Ganuza, Roberto Pino, Francisco Álvarez, José Rengel, Cristina Hernández, Eduardo Uribe, Juan Blanco

**Affiliations:** 1 Facultad de Ciencias del Mar, Departamento de Acuicultura, Universidad Católica del Norte, Larrondo 1281, Coquimbo 1781421, Chile; iranzuganu@gmail.com (I.G.); roberto.pino@alumnos.ucn.cl (R.P.); falvarezsego@gmail.com (F.Á.); jose.rengel@ucn.cl (J.R.); euribe@ucn.cl (E.U.); 2 Centro de Investigación y Desarrollo Tecnológico en Algas (CIDTA), Facultad de Ciencias del Mar, Universidad Católica del Norte, Larrondo 1281, Coquimbo, Chile; mmaraya@ucn.cl; 3 Centro i∼mar & CeBiB, Universidad de Los Lagos, Casilla 557, Puerto Montt 5480000, Chile; patricio.diaz@ulagos.cl; 4 Laboratorio de Biotecnología Aplicada, Facultad de Ciencias Veterinarias, Universidad San Sebastián, Lago Panguipulli 1390, Puerto Montt 5501842, Chile; 5 Centro de Investigaciones Biológicas Aplicadas (CIBA), Diego de Almagro 1013, Puerto Montt 5507964, Chile; 6 Doctorado en Acuicultura, Programa Cooperativo Universidad de Chile, Universidad Católica del Norte, Pontificia Universidad Católica de Valparaíso, Coquimbo 17811421, Chile; 7 Laboratorio Salud Pública, Seremi de Salud Región de Los Lagos, Crucero 1915, Puerto Montt 5505081, Chile; cristina.hernandez@redsalud.gov.cl; 8 Centro de Investigacións Mariñas (Xunta de Galicia), Apto. 13, 36620 Vilanova de Arousa, Pontevedra, Spain; juan.carlos.blanco.perez@xunta.gal

**Keywords:** *Alexandrium catenella*, PSP outbreak, *Mesodesma donacium*, mass mortality, southern Chile

## Abstract

In late February 2016, a harmful algal bloom (HAB) of *Alexandrium catenella* was detected in southern Chiloé, leading to the banning of shellfish harvesting in an extended geographical area (~500 km). On April 24, 2016, this bloom produced a massive beaching (an accumulation on the beach surface of dead or impaired organisms which were drifted ashore) of surf clams *Mesodesma donacium* in Cucao Bay, Chiloé. To determine the effect of paralytic shellfish poisoning (PSP) toxins in *M. donacium*, samples were taken from Cucao during the third massive beaching detected on May 3, 2016. Whole tissue toxicity evidence a high interindividual variability with values which ranged from 1008 to 8763 μg STX eq 100 g^−1^ and with a toxin profile dominated by GTX3, GTX1, GTX2, GTX4, and neoSTX. Individuals were dissected into digestive gland (DG), foot (FT), adductor muscle (MU), and other body fractions (OBF), and histopathological and toxin analyses were carried out on the obtained fractions. Some pathological conditions were observed in gill and digestive gland of 40–50% of the individuals that correspond to hemocyte aggregation and haemocytic infiltration, respectively. The most toxic tissue was DG (2221 μg STX eq 100 g^−1^), followed by OBF (710 μg STX eq 100 g^−1^), FT (297 μg STX eq 100 g^−1^), and MU (314 μg STX eq 100 g^−1^). The observed surf clam mortality seems to have been mainly due to the desiccation caused by the incapability of the clams to burrow. Considering the available information of the monitoring program and taking into account that this episode was the first detected along the open coast of the Pacific Ocean in southern Chiloé, it is very likely that the *M. donacium* population from Cucao Bay has not had a recurrent exposition to *A. catenella* and, consequently, that it has not been subjected to high selective pressure for PSP resistance. However, more research is needed to determine the effects of PSP toxins on behavioral and physiological responses, nerve sensitivity, and genetic/molecular basis for the resistance or sensitivity of *M. donacium*.

## 1. Introduction

Paralytic shellfish poisoning (PSP) is a neurotoxic syndrome caused by the ingestion of shellfish contaminated by saxitoxin and/or its analogues, causing a range of symptoms from slight tingling sensation or numbness around the lips to fatal respiratory paralysis (reviewed by Reference [1]). The toxins involved in this syndrome (paralytic shellfish toxins, PST) are known to be biosynthesized by various species of marine dinoflagellates of the genera *Alexandrium*, *Gymnodinium* and *Pyrodinium* [2,3,4]. PST include more than 57 structurally related compounds from the marine environment [5], which can be grouped into six classes: (a) N-sulfocarbamoyl toxins (GTX5, GTX6, C1–C4), (b) decarbamoyl toxins (dcGTX1–4, dcNeo, dcSTX), (c) carbamoyl toxins (GTX1–4, neoSTX and STX) [6,7], (d) deoxydecarbamoyl (doSTX, doGTX2–3) [6], (e) hydroxybenzoyl toxins (GC1 to GC6) [8,9,10], and other saxitoxin analogues that include 11β-hydroxy-N-sulfocarbamoylsaxitoxin (M1), 11β-hydroxysaxitoxin (M2), 11,11-dihydroxy-N-sulfocarbamoylsaxitoxin (M3), 11,11-dihydroxysaxitoxin (M4), and the unidentified compound (M5) [11].

Some HAB species could affect shellfish, with the responses depending on a series of species-specific or individual characteristics of both phytoplankton (including toxin production) and shellfish [12]. Several behavioral, physiological, and cellular responses of shellfish to toxic *Alexandrium* species have been described, including changes in valve closure, filtration rate, feeding rate, byssus production, oxygen consumption, cardiac activity, neurophysiological effects, and pathological alterations [12,13,14,15,16,17,18,19,20,21,22,23,24,25]. High concentrations of organisms belonging to this genus of dinoflagellates and the persistence of its blooms can produce mass mortalities of shellfish, as those reported from different geographical areas and affecting diverse bivalve species, such as mussel *Mytilus meridionalis,* oyster *Crassostrea virginica*, scallop *Chlamys opercularis*, cockle *Cerastoderma edule,* and clams *Donax serra*, *D. variabilis*, and *Spisula solidissima* [12,26,27,28].

PSP was detected in Chiloé for the first time in 1972, in Bell Bay, Magallanes Region (53°55′ S; 71°45′ W). During that event, three fishermen died due the consumption of the ribbed mussel *Aulacomya atra*, whose toxicity was associated with the presence of the dinoflagellate *Gonyaulax catenella* (currently *Alexandrium catenella*) [29]. Since then, *A. catenella* blooms and PSP toxicity outbreaks have been reported annually covering an extensive area between Magallanes Region to Los Lagos Region, specifically in Quellón, Chiloé Island (43°07′ S; 73°36′ W) [30,31,32], with interannual differences in the affected geographical area and toxicity intensity [33,34,35,36,37,38].

**Figure 1:**
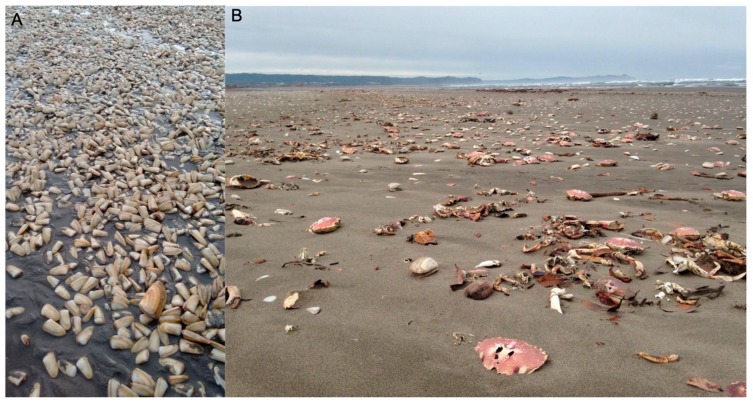
Massive beaching of different invertebrate species recorded in Cucao Bay on May 3, 2016, during an *Alexandrium catenella* toxic bloom.

In 2016, a late summer bloom of *A. catenella* was detected in Southern Chile. This outbreak was the worst event of all those recorded in Chile in terms of geographical extension and affected species, spreading, for the first time, from southernmost of the Chiloé Archipelago (43°50′ S) to Mehuín, Los Ríos Region (39°25′ S) [39], therefore affecting the Pacific Ocean coastal zone. During the toxic episode, the largest invertebrate mass die-off ever recorded in Chile took place. It included mortalities of different organisms, such as the mollusks *Mesodesma donacium* and *Gari solida* and the crustaceans *Austromegabalanus psittacus* and *Romaleon polyodon* (Figure 1). Furthermore, this toxic episode caused the mortality of vertebrates, including sea lions (*Otaria flavescens*), seagulls (*Larus dominicanus)*, and dogs [39]. Human intoxications were also reported (12 people were affected), causing dramatic socioeconomic impacts for more than a thousand fishermen in the affected area [40] because of the banning of shellfish harvesting from the involved natural beds.

*Mesodesma donacium* is an endemic species of the Pacific coast of South America, where it is distributed from Sechura Bay, Perú (5° S) to Chiloé Island, Chiloé (43° S) [41,42], inhabiting oceanic sandy beaches, often located near river mouths, that are characterized by strong waves and highly active sediment dynamics [43]. The populations are confined to the subtidal and intertidal zones, where they are buried in the substrate between 10 to 25 cm depth. Natural beds of this species are distributed along the coast in a patchy way [42,44]. In Chiloé, this is one of the most important commercial species for benthic fisheries, especially in the two most important natural beds located in Coquimbo Bay in northern Chiloé and Cucao Bay, where it is the principal economic resource for local fishermen belonging to the Huilliche ethnic group [45,46].

The aim of the current work was to describe the accumulation of PSP toxins, their distribution and profile in different tissues of *M. donacium*, and their possible link with the massive beaching of this species which took place during a large bloom of *Alexandrium catenella*. 

## 2. Results

### 2.1. Toxicity of Surf Clams and Massive Beaching in Cucao Bay

**Figure 2:**
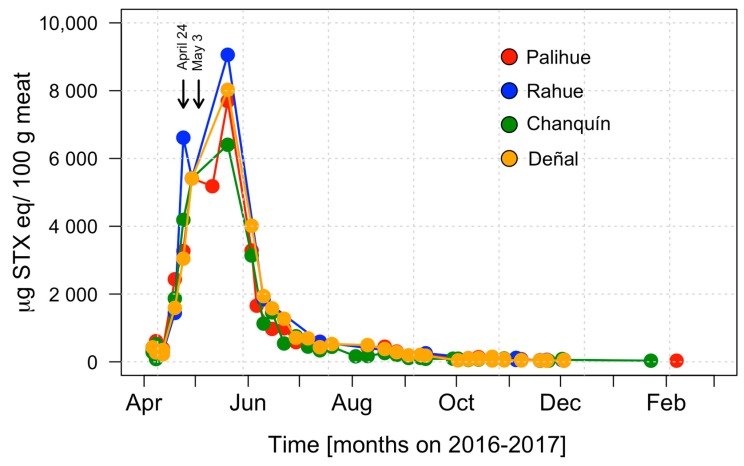
Toxicity changes in surf clams obtained in different locations from Cucao Bay during the March 2016–January 2017 toxic outbreak. Arrows indicates massive beaching on April 24 and May 3, 2016.

**Figure 3:**
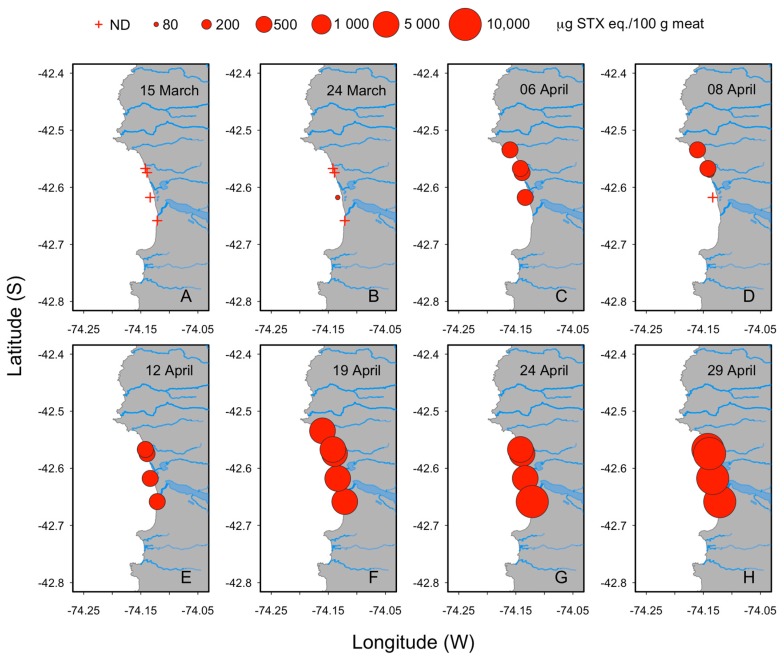
Temporal and spatial distribution of paralytic shellfish poisoning (PSP) toxicity in *M. donacium* along the coast from Cucao Bay during toxification phase of shellfish. (**A**) March 15th, (**B**) March 24th, (**C**) April 6th, (**D**) April, 8th, (**E**) April 12th, (**F**) April 19th, (**G**) April 24th and (**H**) April 29th.

The analysis of surf clam samples collected from Cucao Bay revealed the occurrence of a PSP toxic episode with toxicities that increased rapidly during the first three sampling weeks (Figure 2). The beginning of the episode (Figure 3) was detected on March 24, 2016, in Chanquín with a toxicity of 33 μg STX eq 100 g^−1^. Two weeks later on April 6, an abrupt increase of toxicity was detected in all localities reaching values of 394, 351, 428 μg STX eq 100 g^−1^ for Chanquín, Palihue and Deñal, respectively, that exceeded the regulatory limit (80 μg STX eq 100 g^−1^). These toxicity levels remained stable for one week and then began to rise quickly, reaching toxicities higher than 1400 µg STX eq 100 g^−1^ (1869, 2436, 1591, and 1442 μg STX eq 100 g^−1^ for Chanquín, Palihue, Deñal, and Rahue, respectively). On April 24, the toxicity increased again to reach values between 3044 and 6614 μg STX eq 100 g^−1^. This increase was followed by the first massive beaching, which covered most Cucao beach (5 km long). At the end of April, the toxicity increased again to reach values near 5500 μg STX eq 100 g^−1^ in all localities and a second massive beaching was recorded. These toxicity levels remained approximately stable for 10 days from May 1 to May 11, a period during which at least three new surf clam beaching events were detected. The peak of shellfish toxicity was observed at May 20th, with values reaching 9059 μg STX eq 100 g^−1^ (6404, 7692, 8026, and 9059 μg STX eq 100 g^−1^ for Chanquín, Palihue, Deñal, and Rahue, respectively (Figure 2 and Figure 3).

Since June 3, the toxicity decreased quickly to levels between 3137 and 4014 μg STX eq 100 g^−1^. One week later, the toxicity additionally decreased to values below 2000 μg STX eq 100 g^−1^. Since then, the toxicity declined gradually to 500 μg STX eq 100 g^−1^ by the end of July, and to 80 μg STX eq 100 g^−1^ (the regulatory limit) by October 29. On January 23, 2017 PSP was only detected in Chanquín (33 μg STX eq 100 g^−1^). 

### 2.2. Visual Observations of Surf Clams during Massive Beaching

On April 24, a seafood inspector of Laboratorio de Salud Pública Ambiental detected the first massive beaching of *M. donacium* covering most of Cucao beach. During this episode, thousands of individuals were found dead, dying, or paralyzed, lying on the sand surface. Two days later, the inspectors reported a notable decrease in the beaching surf clams, estimating that only 20% of individuals were lying on the sand. On May 3, during a new beaching episode, the density of surf clams on the sand surface was 40–50 individuals m^−2^. Most of the specimens lying on the surface were alive with the foot or siphons extended or with the valve partially closed. A detailed examination revealed that the individuals were paralyzed because they had a weak reaction to mechanical stimulus on foot or siphons, with a slow and incomplete retraction. Three days later, the beaching surf clams decreased again, suggesting that some of the alive individuals recover the ability to re-burrow in the sand.

### 2.3. Toxicity and Toxin Profile of Whole Individuals

**Figure 4:**
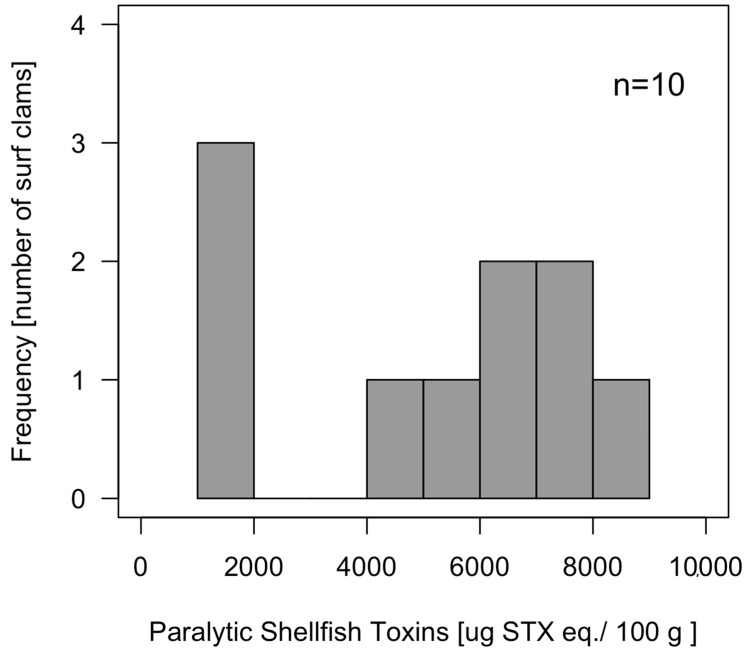
Interindividual toxicity of *M. donacium* obtained from Cucao Bay on 3 May 2016.

**Figure 5:**
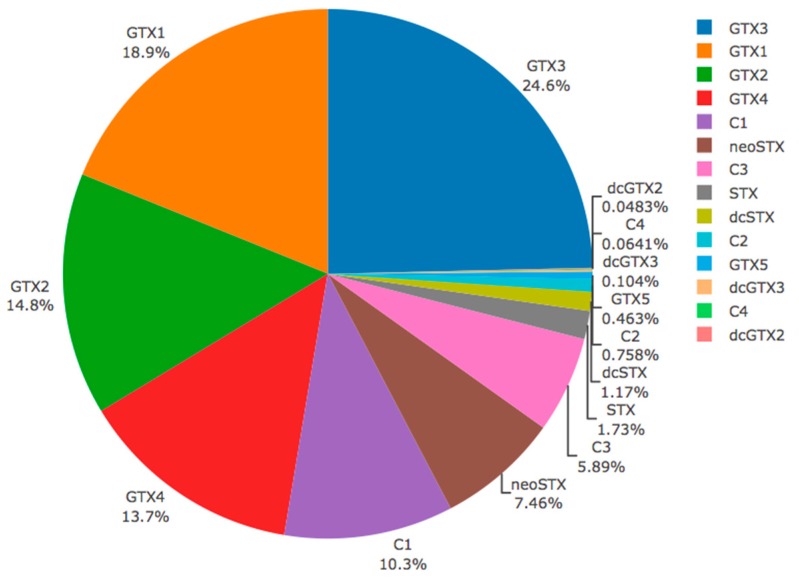
Relative toxin profile (% mole) of whole individuals of *Mesodesma donacium* collected from Cucao Bay (*n* = 10).

The analyses of individual surf clams obtained in Chanquín on May 3, 2016 revealed a high toxicity with values between 1008 to 8763 μg STX eq 100 g^−1^ (4699 ± 2971 μg STX eq 100 g^−1^), showing a bimodal distribution (Figure 4) with no individual with toxicities between 2000 and 4000 μg STX eq 100 g^−1^. The toxin profile (% mole), was dominated by carbamoyl toxins that, in decreasing order of concentration, were GTX3 (24.64%), GTX1 (18.88%), GTX2 (14.79%), GTX4 (13.70%), neoSTX (7.46%), and STX (1.73%). Other toxins detected in less amount were N-sulfocarbamoyl, such as C1 (10.32%), C3 (5.89%), C2 (1%), and C4 (0.06%), and decarbamoyl, dcSTX being (1.17%) the most abundant toxins of this group, with dcGTX2 and dcGTX3 present in trace levels (<1%) (Figure 5).

### 2.4. Toxicity and Toxin Profile of Surf Clam Tissues

**Figure 6:**
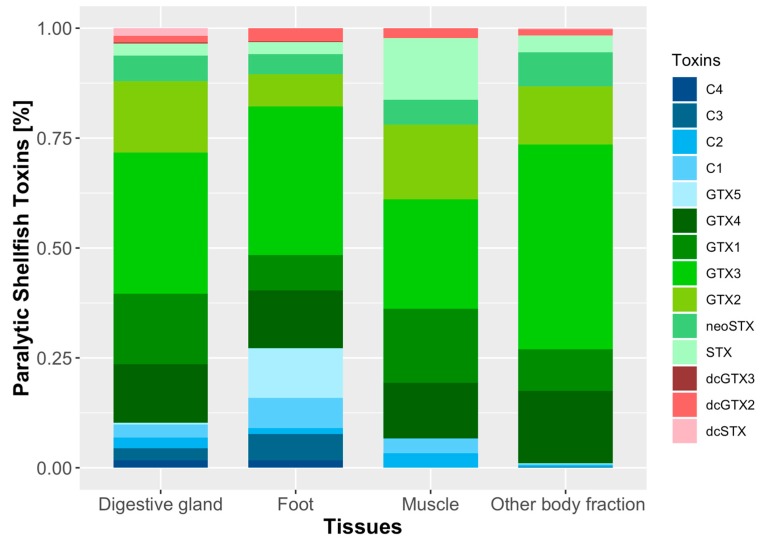
Relative toxin profile (% mole) of different organs of *Mesodesma donacium* collected from Cucao Bay (*n* = 10).

The most toxic organ was the digestive gland (DG), with 2221 ± 2193 μg STX eq 100 g^−1^. On a molar basis, the toxin profile was dominated by carbamoyl toxins, GTX3 being (32.1%) the most abundant, followed by GTX2 (16.2%), GTX1 (16.1%), GTX4 (13.3%), neoSTX (5.9%), and STX (2.74%). The second group in importance was that of N-sulfocarbamoyl toxins, with C1, C3, C2, C4, and GTX5, in decreasing order of abundance. The less abundant of the analyzed toxins were dcGTX3 and dcGTX2 (Figure 6, Figure S1B).

The toxicity of all other body fractions (OBF) was the second in importance being approximately 1/3 as toxic as DG (710 ± 450 μg STX eq 100 g^−1^). The toxin profile was dominated mainly by carbamoyl toxins (GTX3 46.5%, GTX4 16.4%, GTX2 13.3%, GTX1 9.5%, neoSTX 7.8%, and STX 3.8%). In this case, the contribution of N-sulfocarbamoyl and decarbamoyl toxins were less relevant to the toxin profile (Figure 6).

Foot (FT) had about 1/8 the toxicity of DG, with values of 297 ± 157 μg STX eq 100 g^−1^. In this tissue, the toxin profile was dominated by carbamoyl toxins (GTX3 33.8%, GTX4 13.1%, GTX1 8%, GTX2 7.4%, neoSTX 4.6%, and STX 2.8%). Another relevant group was that of N-sulfocarbamoyl toxins, such as GTX5 (11.3%), C1 (6.9%), and C3 (6%). The toxins of the decarbamoyl group dcGTX2–3 were present in a very low molar percentage (Figure 6, Figure S1C).

Finally, muscle (MU) had a toxicity of 314 ± 8.5 μg STX eq 100 g^−1^. Its toxin composition (in molar proportion) was dominated by carbamoyl toxins (GTX3 24.8%, GTX2 17.1%, GTX1 16.9%, GTX4 12.6%, STX 14%, and neoSTX 5.6%), followed by other toxins present in low molar percentages as C1, C2, and dcGTX2 (Figure 6, Figure S1D).

### 2.5. Surf Clam Histology

**Figure 7:**
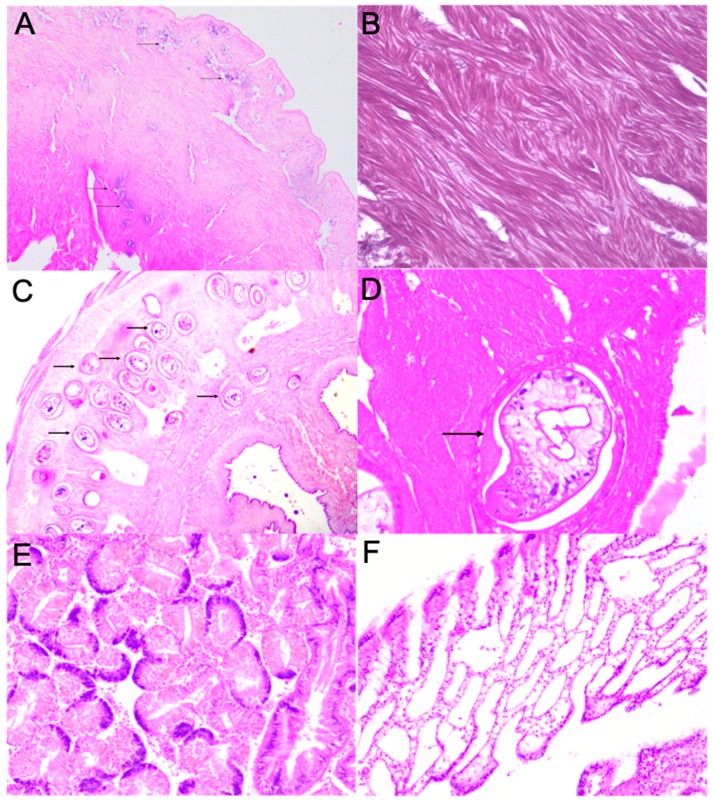
Histophatological section of *M. donacium* (H&E, medium magnification): (**A**–**B**) Foot; (**C**–**D**) Siphon; (**E**) Gill; and (**F**) Digestive gland. Cysts of digeneans metacercaria (arrows).

Some pathological conditions were observed in *M. donacium* tissues exposed to the HAB of *A. catenella*. Bacterial colonies were found in the foot (Figure 7A), mantle, and muscle (with bacillary form in foot and mantle) in 40% of individuals. Additionally, cysts of digeneans metacercaria in the siphon muscle (Figure 7C, D) were found in all the analyzed individuals. Hemocyte aggregation in gill filaments was detected in 50% of individuals (Figure 7E) and haemocytic infiltration of the connective tissue surrounding the digestive gland tubules (Figure 7F) in 40%. No significant pathological changes were found in the foot (Figure 7B).

## 3. Discussion

During the bloom, *M. donacium* quickly bioaccumulated PSP toxins up to a maximum toxicity of 9059 μg STX eq 100 g^−1^, which is 113 times the level considered hazardous for human consumption. This was the highest level ever recorded in this species since the monitoring program begun in 1995, attesting the magnitude and persistence of the toxic *A. catenella* bloom. The toxicity detected in *M. donacium* is higher than that found in other clams from southern Chile, such as *Venus antiqua* (111 μg STX eq 100 g^−1^) and *Tagelus dombeii* (262 μg STX eq 100 g^−1^), and similar to those reported in *Gari solida* (3286 μg STX eq 100 g^−1^) [47]. However, the toxicity was lower than that reported in the Chilean blue mussels *Mytilus chilensis* 22,000 μg STX eq 100 g^−1^ [48], ribbed mussels *Aulacomya atra* in 1996 (113,259 μg STX eq 100 g^−1^) [49], and recently, in 2018, when a global record of 143,000 μg STX eq 100 g^−1^ in *M. chilensis* [50] was attained.

Toxicity of the clams obtained during the third massive beaching ranged from 1008 to 8763 μg STX eq 100 g^−1^ (average 4699 μg STX eq 100 g^−1^), revealing a high interindividual variability (CV = 67%) in PST bioaccumulation. This interindividual variation is similar to that reported in other shellfish, such as the clams *Arctica islandica* (56%) [51], *Spisula solidissima* (49%), and the scallop *Placopecten magellanicus* (44%) [51,52], but is lower than those reported in the clam *Panopea abrupta* (93%) [53]. The interindividual variability could be explained by diverse physiological processes, that, in turn, could be affected by the sensitivity of shellfish to PST toxins [54]. Diverse studies compared feeding behavior and physiology of different bivalve species, showing that the feeding response was correlated with the animal sensitivity to toxins and to the algal toxicity. For example, the clams *Mya arenaria*, *Ruditapes philippinarum*, and *Tagelus dombeii* and the oysters *Crassostrea virginica* and *Magallana gigas (Crassostrea gigas*) decrease the clearance rate on the presence of the toxic dinoflagellate *Alexandrium tamarense* [15,55,56,57,58,59]. The causes of the variability of toxin accumulation at interindividual level, notwithstanding, have been much less studied, but studies developed in *M. gigas* suggest that the feeding behavior is mainly responsible for interindividual variation in toxin bioaccumulation [54,60,61].

In the whole tissues of *M. donacium*, the toxin profile (in decreasing order) was dominated by GTX3, GTX1, GTX2, GTX4, C1, neoSTX, and C3, which contribute 95% of the total toxin content, while the remaining 5% corresponded to STX, dcSTX, C2, GTX5, C4, dcGTX2, and dcGTX3. The toxin profile is similar to those reported in the ribbed mussel *Aulacomya atra* from Darwin Channel, southern Chiloé (but differ in its higher proportion of GTX5) [49] and *M. chilensis* from Errazuriz Channel, Magallanes region, dominated mainly by GTX2 [62]. Similar profiles were found in the clams *Venus antiqua* and *Tagelus dombeii* (dominated by gonyautoxins, neoSTX and STX), but differ by the absence of C toxins [47]. Unfortunately, there is no information related to the toxic profile of *Alexandrium catenella* from Cucao Bay. The profile of *M. donacium*, however, showed characteristics which are very similar to that of strains of *A. catenella* isolated in other episodes, as the clones PFB36 from San Pedro Island (Los Lagos) and ACC02 from Coastal Channel (Aysén region). In both cases, the profile mainly differs by the presence of GTX6 in the dinoflagellates [33,63].

In the beached *M. donacium*, all tissues had a toxicity higher than the regulatory limit (80 μg STX eq 100 g^−1^). In this shellfish, the relative contribution of the different tissues to toxicity (expressed as % μg STX eq) had the following pattern: digestive gland (DG) (68.4%), foot (FT) (23.7%), other body fractions (OBF) (7.4%), and MU (0.4%). In general, the anatomical distribution of toxicity in *M. donacium* is similar to those reported in other shellfish, in which DG is the initial repository of PST toxin during the intoxication phase [57]. Some examples of PST preferential accumulation in DG are the clams *Mya arenaria* (89.3%) [57], *Spisula solidissima* (85.6%) [64], *Mercenaria mercenaria* (80.9%) [65], and *Saxidomus giganteus* (66.2%) [57]. The FT tissues of *M. donacium* had a high contribution to toxicity (23.71%) as compared with the clam species mentioned above, in which this tissue only contributes 1.4 to 2.2% [57]. All tissues studied have a toxin profile similar to that of whole individuals in which the profile was dominated by GTX3, GTX2, GTX4, GTX1, neoSTX, and STX that contribute 97.3, 91.1, 86.2, and 69.6% for OBF, MU, DG, and FT, respectively. The most remarkable differences in toxin profile were observed in FT tissues with high contribution of GTX5 (11.3%), C1 (6.7%), C3 (6.0%), and dcGTX2 (3%). In this tissue, the high percentage of GTX5 suggests that a possible transformation of these toxin to STX by hydrolysis could be slower in comparison to the other surf clam tissues that have a low proportion of GTX5 (e.g. DG and OBF). Conversion of GTX5 to STX by hydrolysis was described in few bivalves as the mussel *Mytilus edulis* [66] and the clam *Paratapes undulatus* [67]. The high percentage α-isomers (C1, C3 in all tissues, and GTX1 in DG and MU) suggest other plausible transformation in *M. donacium* that corresponds to epimerization. In this case, the β-epimers (C2, C4, GTX3, and GTX4) will gradually convert into their thermodynamically more stable forms that correspond to α-isomers (C1, C3, GTX2, and GTX1) [6]. Epimerization is the most common bioconversion found in the bivalve tissues and has been described in the scallop *Pecten novaezelandiae* [68], mussels *M. edulis* [69] *M. galloprovincialis* [70,71], and the clam *Panopea globosa* [72]. Finally, the presence of dcGTX2, dcGTX3 (except in MU), and dcSTX (except in FT and MU) suggests the capability of *M. donacium* tissues to transform carbamoyl toxins GTX2, GTX3, and STX to their corresponding decarbamoyl derivatives and was demonstrated in clams *Protothaca staminea* [73], *Spisula solida* [74], *Mactra chinensis*, and *Perodina venulosa* [6]. 

Exposure to *A. catenella* seems to have induced immunological and inflammatory responses in *M. donacium*. Histopathological analyses in different tissues of the individuals exposed to the *A. catenella* bloom revealed the presence of hemocyte aggregation in gill tissues of 50% of analyzed individuals, that could be associated with an immunological response to *A. catenella* cells during filtration processes. In bivalves, the gill is one of the most important organs because of it is involved in respiration and feeding, being the first organ that has contact with toxic phytoplankton cells [75]. As in *M. donacium*, Estrada et al. [76] observed hemocyte aggregation in gill of the scallop, *Nodipecten subnodosus* exposed to the dinoflagellate PSP-producing *Gymnodinium catenatum*.

Inflammatory responses, as the observed haemocytic infiltration in connective tissues of digestive gland or other similar ones as diapedesis of hemocytes, were described in *Mytilus edulis, M. gigas*, and *Nodipecten subnodosus* exposed to *A. fudyense*, *A. minutum*, and *G. catenatum* [75,76,77] (all of them being PSP producers). Haberkorn et al. [75] proposed three hypotheses to explain inflammatory responses in this organ. The first suggested that the massive migration of hemocytes into the lumina of the digestive gland is a defense response of bivalves to protect tissues from toxicity or to remove toxic cells [77,78]. The second hypothesis proposed that hemocyte migration is a response to opportunistic bacterial infections that could appear by the exposure to toxic algae [77,78]. The third hypothesis suggested that the hemocyte diapedesis across intestine epithelia could be considered as a detoxification pathway [79].

In some individuals of beached surf clams, bacterial colonies were detected in the muscle, foot, and mantle and could be attributed to opportunistic bacteria of vibrio type. Abi-Khalil et al. [80] demonstrated that exposure to the neurotoxic *Alexandrium catenella* increases the susceptibility of *M. gigas* oysters to the pathogenic *Vibrio tasmaniensis* LGP32. In addition, they suggested that PSP toxins alone are not sufficient to induce mortalities but could rather participate in the induction of oyster mortality in a multifactorial way that involves vibrios and likely another pathogen.

The presence of cyst of digeneans metacercaria in siphon muscle is common in *M. donacium*, so it is not possible to infer if the *A. catenella* bloom had intensified the severity of infection or the impact on the bivalve population. López et al. [81] described a high prevalence of species belonging *Monorchiidae* family in bivalves obtained from different sites of Los Lagos region, including Cucao Bay. Lassudrie et al. [24], notwithstanding, suggested that the oyster *M. gigas* was more susceptible to trematode infestations when exposed *Alexandrium* blooms, which can produce degeneration of muscle fiber that could compromise valve-closure movements and the contraction of the adductor muscle.

During the bloom of *A. catenella*, a large number of individuals of *M. donacium* were either dead, dying, or paralyzed, lying on the sand surface. The observed reduction in foot contraction and valve gape and siphon retraction suggests a loss of nervous control due to PSP toxins. In *M. donacium*, the ability to re-burrow of some individuals from Cucao Bay, together with high interindividual variability (8-fold) and the bimodal distribution of toxin concentration, suggest the presence of two groups of individuals, one of them sensitive to the toxins (incapable of burrowing and with low toxin accumulation capability), and another one (more) resistant to the toxins (capable of burrowing and with high toxin accumulation capability), as described in *M. arenaria* [82,83]. PSP toxins can adversely affect susceptible marine invertebrates by blocking the Na^++^ influx in excitable cells and thus inhibiting the nerve action potential, leading to paralysis and death [84]. Indeed, the burrowing incapacitation produced by PSTs was described in the clam *Mya arenaria*, in which differences were found between resistant individuals, from populations frequently exposed to toxic *Alexandrium* blooms (Bay of Fundy, Canada), and sensitive individuals from areas with no record of PSP toxic episodes (St Lawrence Estuary, Canada) [15,85]. Considering the available information of the monitoring program and taking into account that this episode was the first detected along the open coast of the Pacific Ocean in southern Chiloé, it is very likely that the *M. donacium* population from Cucao Bay has not had a recurrent exposition to *A. catenella* and consequently that it has not been subjected to high selective pressure for PSP resistance. MacQuarrie and Bricelj [82] found high differences in toxicity accumulation in a laboratory experiment with *M. arenaria*, reporting a maximum toxicity in resistant individuals (77,000 μg STX eq 100 g^−1^) 10-fold higher than the toxicity in sensitive ones (8200 μg STX eq 100 g^−1^).

Our observations in this study suggest that the surf clam mortality during *A. catenella* toxic bloom in Cucao Bay was mainly due to incapability of burrowing—an indirect effect of PSP toxin-induced paralysis—which caused desiccation. However, more research is needed to determine the effects of PSP toxins on behavioral and physiological responses, nerve sensitivity, and genetic/molecular basis for the resistance or sensitivity of *M. donacium*.

## 4. Materials and Methods

### 4.1. Study Area

**Figure 8:**
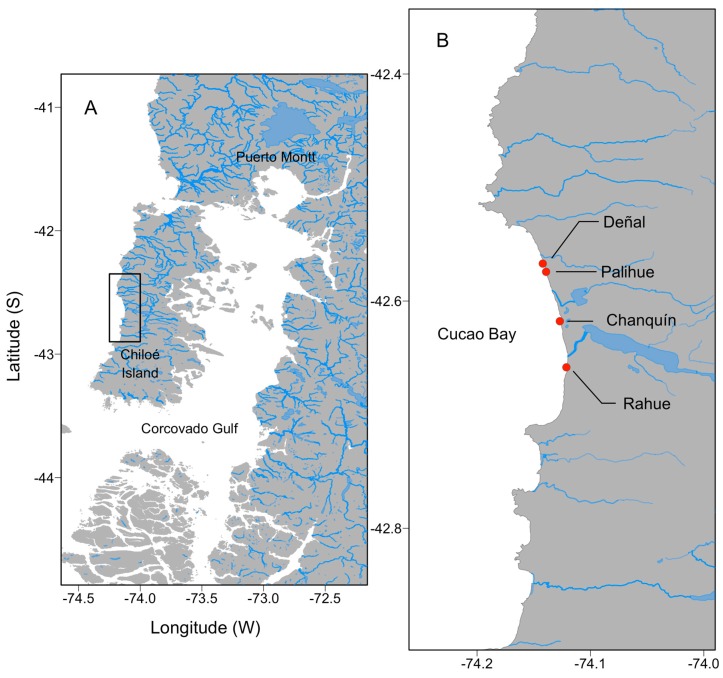
Study area in southern Chiloé showing (**A**) The map shows a section of the Chilean Inland Sea; (**B**) The four sampling stations at Cucao Bay in the oceanic coast of the Chiloé Island.

Cucao Bay (42°38′ S–74°07′ W) is located in the oceanic coast of the Chiloé Island (Figure 8). This site is characterized by the presence of large natural bed of surf clams (*M. donacium*) along a 20 km sandy beach. In general, this system is characterized by strong oceanic water influence, with salinity >31 despite the freshwater inputs from the Cucao River and other smaller tributaries (Figure 2). Water temperature ranges from 10 to 14 °C (IFOP, unpublished data) and the area is subjected to semidiurnal tides with amplitudes ranging from 2 m (neap tides) to 4 m (spring tides) (www.shoa.cl).

### 4.2. Shellfish Sampling, Toxin Extraction, and Biological Method Analyses Used for Rutine Monitoring

Within the framework of the regional monitoring program developed by the Laboratorio de Salud Pública Ambiental (Secretaría Regional Ministerial de Salud, Region de Los Lagos), surf clam samples were obtained periodically from Cucao Bay at least twice a month—from March 15, 2016, to January 23, 2017—in four sampling stations located at Cucao Bay: Deñal, Palihue, Chanquín, and Rahue (Figure 8).

Each sample consisted of at least 20 individuals of commercial size (>6 cm) collected by fishermen by means of hang-gathering. Samples were placed inside a plastic bag, then placed in a cool box at 10 °C and transported to the laboratory. Upon arrival to the laboratory, paralytic shellfish toxins (PST) were extracted from surf clam tissues following the official AOAC method 959.08 [86]. For toxin extraction, 100 g of homogenized raw tissues were mixed with 100 mL of HCl (0.1 N) solution using a blender and then boiled for 5 min. The sample was cooled at room temperature for 10 min, then the pH was corrected to between 2–4. At the end of the procedure, the resulting extract was transferred to a 200 mL volumetric flask and filled up to the mark with HCl (0.003 N). Aliquots (1 mL) of the final extract were intraperitoneally injected into three Swiss mice weighing 19–21 g following the official AOAC method 959.08, and their death times were recorded. If any mouse died in less than 5 min, the test was performed again using diluted samples until the mouse died within 5 to 7 min. The toxicity was calculated and expressed as μg STX eq 100 g^−1^ sample using Sommer’s Table.

### 4.3. Shellfish Sampling, Toxin Extraction, Chromatographic, and Histopathological Analyses during Beaching

Data: Surf clam samples were collected from Bahía Cucao during a massive beaching detected on May 3, 2016. The sample consisted of 20 individuals of commercial size (>6 cm) collected from the surface of the sand. To ensure that the shellfish were still alive, a mechanical stimulus was induced by means of a puncture in the foot using a steel needle to determine foot contraction, incapability, or reduction of valve closure and siphon retraction. Upon arrival to the laboratory, the shellfish were washed and distributed into two subsamples completely at random. The first subsample consisted of 10 clams and was used for individual whole sample analyses. The second subsample contained 10 individuals and was used to determine the anatomical distribution. These individuals were carefully dissected into foot (FT, the edible tissue) and the nonedible tissues corresponding to digestive gland (DG), adductor muscle (MU), and all other body fractions (OBF) (including mantle, gill, kidney, and siphons). Each tissue was extracted following the official AOAC method 2011.02 [87], with slight modifications. Extraction was performed with HCl (0.1 N) (1:1, *w/v*) using an Ultra-Turrax T25 dispersing system (IKA® Werke GmbH & Co. KG, Staufen, Germany) at 11,000 rpm for 3 min. After each extraction, the pH of the resulting emulsion was adjusted to 2–4, boiled at 100 °C for 5 min, centrifuged at 5000 *g* for 20 min (Centurion K2015R, Centurion Scientific Ltd, Stoughton, West Sussex, UK), and its pH adjusted again to 2–4. To deproteinate the samples, a 1 mL aliquot was mixed with 50 μL of 30% trichloroacetic acid (TCA), vortex mixed, and then centrifuged at 9000 *g* for 5 min. To neutralize the solution, 70 μL of NaOH (1 M) was added, and then vortexed and centrifuged at 9000 *g* for 5 min. One 500-μL aliquot was filtered through a 0.2 μm Clarinert nylon syringe filter (13 mm diameter) (Agela technologies, Torrence, CA, USA) and stored into an autosampler vial. A second 500-μL aliquot was hydrolyzed in order to transform the sulfocarbamate toxins (if present) to the corresponding carbamate toxins (HCl 0.4 N, 100 °C, 15 min), filtered through 0.2 μm nylon filter, and stored into an autosampler vial. For some analyses, extracts were diluted 5-fold or 10-fold with HCl 0.1 N to ensure the correct quantification of the most abundant toxins in the samples.

The most of important PST toxins were quantified following the official AOAC method 2011.02 by high performance liquid chromatography (HPLC) using a Hitachi LaChrom Elite HPLC system equipped with a Hitachi FL detector L2485 (Hitachi High Technologies America Inc., Chatsworth, CA, USA) and a post-column reaction system composed by two Waters pumps (Water Reagent Manager) and a 20 m Teflon coil (2 mL) in a heating reactor (Pickering Laboratories, Mountain View, CA, USA). The chromatographic separation was carried out using a Zorbax Bonus RP column (150 × 4.6 mm, 3.5 μm particle diameter) (Agilent, Santa Clara, CA, USA) with a Zorbax Bonus RP guard column (12.5 × 4.6 mm, 5 μm particle diameter) at 40 °C. For the separation, two mobile phases were used consisting of 11 mM heptanesulfonate and 5.5 mM phosphoric acid solution adjusted to pH 7.1 with ammonium hydroxide (A) and 11 mM heptane sulfonate, 16.5 mM phosphoric acid, 11.5% acetonitrile solution adjusted to pH 7.1 with ammonium hydroxide (B). A gradient elution started with a proportion of 100% A, which was maintained for 8 minutes, followed by a linear increase to 100% B from minute 8.01 to minute 15, and then held for 4 min. The column was equilibrated in the initial conditions for 5 min previously to next run. The mobile phase flow was 0.8 mL min^−1^ and the injection volume 10 μL. After separation, the toxins were derivatized in the post-column reaction system (85 °C) with a solution of 100 mM phosphoric acid, 5 mM periodic acid solution adjusted to pH 7.8 with 5 M sodium hydroxide, and, at the end of the reaction coil, mixed with a solution of 0.75 M nitric acid. The flow rate of both solutions was 0.4 mL min^−1^. Finally, the detection of the fluorescent derivatives of the toxins was carried out with an FL detector set to 330/390 nm excitation/emission wavelengths. PST concentration in the samples was quantified by comparing the obtained response with that of corresponding reference materials (from IMB-NRC, Ottawa, ON, Canada). The limits of detection of the technique were estimated for a signal/noise ratio of 3 (Table S1). Finally, to determine the total toxicity for each sample, equivalency factors (TEFs) were used for calculations [88].

Upon arrival to the laboratory, 10 individuals were carefully dissected for histopathological studies in siphons (SI), gill (GI), gonad (GO), digestive gland (DG), foot (FT), and mantle (MA). Each tissue sample was placed in a cassette, immediately fixed in Davidson´s solution [89] and kept at 4 °C for 48 h before being switched to ethanol (70%). The tissue samples were then dehydrated in ascending ethanol solutions, cleared with xylene, and embedded in paraffin wax using a spin tissue processor (STP 120, Microm International GmBH Thermo Scientific, Walldorf, Germany). Finally, 5 μm thick sections of each tissue were obtained using a rotary microtome (Microm HM325, Microm International GmBH Thermo Scientific, Walldorf, Germany), mounted on slides and stained with Harry´s hematoxylin and eosin [90]. All sample tissues were examined under a light microscope (Leica DFC295) and micrographs of the material were obtained by means a microscope camera (Leica DM 2000 LED). 

## Supplementary Materials

The following are available online at https://www.mdpi.com/2072-6651/11/4/188/s1, Figure S1: Selected chromatograms of Paralytic Shellfish Toxins (PST) in reference material (**A**), digestive gland (**B**), foot (**C**) and muscle (**D**), Table S1: Limits of detection (LOD) and quantification (LOQ) for each toxin in *Mesodesma donacium*.
