# Supplementary material for: Paralytic Shellfish Toxins in Surf Clams Mesodesma donacium during a Large Bloom of Alexandrium catenella Dinoflagellates Associated to an Intense Shellfish Mass Mortality
Source: Toxins (Basel). 2019 Mar 29;11(4):188. doi: 10.3390/toxins11040188 (PMC6520680; doi:10.3390/toxins11040188)
Supplement: Supplementary file 1 [file toxins-11-00188-s001.pdf]

Supplementary Material

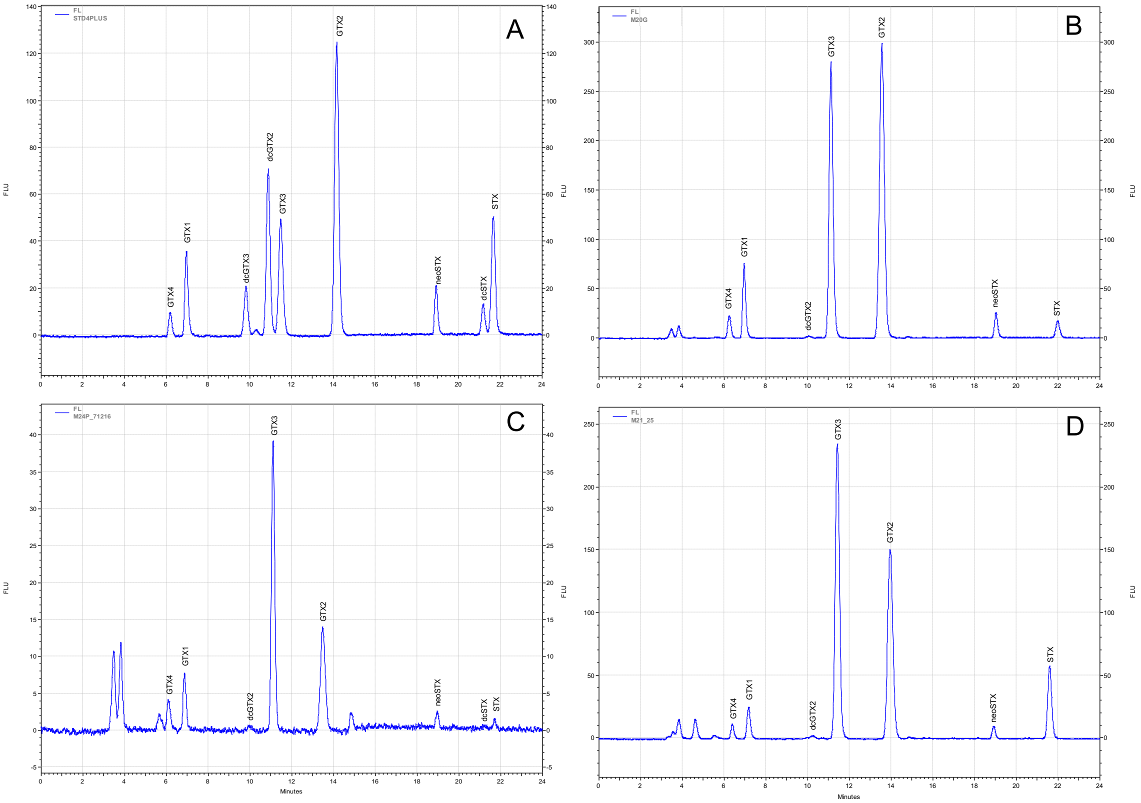

S1. Selected chromatograms of Paralytic Shellfish Toxins (PST) in reference material (A), digestive gland (B), foot (C) and muscle (D).

S2. Limits of detection (LOD) and quantification (LOQ) for each toxin in *Mesodesma donacium*

TOXIN	LOD<br>mg STX diHCl kg^{-1}	LOQ<br>mg STX diHCl kg^{-1}
**GTX4**	0.031	0.09
**GTX1**	0.055	0.17
**dcGTX3**	0.005	0.01
**dcGTX2**	0.005	0.02
**GTX3**	0.009	0.03
**GTX2**	0.003	0.01
**NEO**	0.042	0.13
**dcSTX**	0.051	0.15
**STX**	0.017	0.05
